# Using individual networks to identify treatment targets for eating disorder treatment: a proof-of-concept study and initial data

**DOI:** 10.1186/s40337-021-00504-7

**Published:** 2021-11-04

**Authors:** Cheri A. Levinson, Rowan A. Hunt, Ani C. Keshishian, Mackenzie L. Brown, Irina Vanzhula, Caroline Christian, Leigh C. Brosof, Brenna M. Williams

**Affiliations:** Department of Psychological and Brain Sciences, University of Louisville, Life Sciences Building, Louisville, KY 40292 USA

**Keywords:** Eating disorders, Network analysis, Personalized treatment, Idiographic modeling, Precision treatment

## Abstract

**Background:**

Eating disorders (EDs) are severe mental illnesses, with high morbidity, mortality, and societal burden. EDs are extremely heterogenous, and only 50% of patients currently respond to first-line treatments. Personalized and effective treatments for EDs are drastically needed.

**Methods:**

The current study (N = 34 participants with an ED diagnosis collected throughout the United States) aimed to investigate best methods informing how to select personalized treatment targets utilizing idiographic network analysis, which could then be used for evidence based personalized treatment development. We present initial data collected via experience sampling (i.e., ecological momentary assessment) over the course of 15 days, 5 times a day (75 total measurement points) that were used to select treatment targets for a personalized treatment for EDs.

**Results:**

Overall, we found that treatment targets were highly variable, with less than 50% of individuals endorsing central symptoms related to weight and shape, consistent with current treatment response rates for treatments designed to target those symptoms. We also found that different aspects of selection methods (e.g., number of items, type of centrality measure) impacted treatment target selection.

**Conclusions:**

We discuss implications of these data, how to use idiographic network analysis to personalize treatment, and identify areas that need future research.

*Trial registration***:** Clinicaltrials.gov, NCT04183894. Registered 3 December 2019—Retrospectively registered, https://clinicaltrials.gov/ct2/show/NCT04183894. NCT04183894 (ClinicalTrials.gov identifier).

**Supplementary Information:**

The online version contains supplementary material available at 10.1186/s40337-021-00504-7.

## Plain English Summary

Eating disorders are severe psychiatric illnesses that carry high mortality, morbidity, and societal and personal burden. Treatments for eating disorders only work in 50% of patients, signifying a great need to improve treatments. One reason that treatments may not work, is because eating disorders vary substantially from one individual to the next, which existing treatments do not fully consider. The current study (N = 34 participants with an eating disorder diagnosis) uses a new modeling technique to identify which symptoms should be targeted in treatment in a personalized manner. As expected, we found that, using this modeling technique, symptoms that should be targeted in treatment vary considerably. We discuss how to use this modeling technique to identify individual treatment targets and ways in which the field can use this strategy to improve existing and create new treatments.

**Supplementary Information:**

The online version contains supplementary material available at 10.1186/s40337-021-00504-7.

## Background

Eating disorders (EDs) are extremely debilitating and carry high impairment and societal costs [3, 10]. In 2018–2019 alone, the United States spent over $64.7 billion on EDs, and 10,000 individuals died prematurely because of an ED [10]. Despite the high cost, mortality, and prevalence associated with EDs, treatments are suboptimal [50]. Response rates for gold standard treatments, such as Enhanced Cognitive Behavioral Therapy (CBT-E; [15], only yield 50% response rates and up to 35% of those who remit will eventually relapse [7, 31, 32, 48]. New treatments, especially personalized treatments, are urgently needed.

EDs are highly heterogenous, both in terms of symptom presentation and treatment response [30, 36]. Almost half of individuals with an ED diagnosis meet criteria for other specified feeding and eating disorder (OSFED, any clinical ED that does not specifically meet criteria for ED diagnostic categories of anorexia nervosa (AN), bulimia nervosa (BN), or binge eating disorder (BED; [21]. Additionally, even within a similar AN diagnosis, symptom presentation is drastically different [36]. For example, one individual with AN may restrict, be afraid of high calorie foods, and meet criteria for an anxiety disorder, whereas another individual may purge, be fearful of interoceptive sensations, and meet criteria for depression. Practically, this heterogeneity means treatments developed based ‘on average symptom presentation’ will fail to effectively treat symptoms for a large majority of individuals. Recent research supports this idea, showing that while about half of individuals with EDs demonstrate symptoms related to overvaluation of weight and shape as most important symptoms for maintenance of psychopathology, about half endorse other non-weight and shape symptoms as most important (Levinson et al., 2021). Accordingly, it is unsurprising that at least 50% of patients do not respond to standard treatments, such as CBT-E, designed to target reduction of ED behaviors and overvaluation of weight and shape applied in a similar order and manner for each patient. In response to this high heterogeneity, clinicians generally adapt evidence-based treatments based on clinical judgment, despite the fact that clinician judgment is flawed [22]. This means there are two barriers to delivering effective ED care, high heterogeneity and the reliance on clinician-judgment. Ideally, clinicians would have a data-driven method to support treatment target selection and guide treatment planning, such that reliance on clinician or treatment team-judgment could be minimized and based on data. Unfortunately, no such method to guide clinicians on treatment selection currently exists.

To address the problematic treatment response rates both for EDs and other psychiatric illnesses, researchers and granting institutions (e.g., National Institutes of Health) have launched the precision medicine initiative [28], which calls for the development and implementation of precision-based treatments that use data to guide treatment development and planning on the individual level [51]. One area that has been proposed as a method to develop personalized treatments is idiographic network analysis (NA; [42, 43]. Idiographic NA utilizes intensive longitudinal data, generally collected via ecological momentary assessment (EMA: a type of intensive assessment methodology, generally delivered to a phone or tablet, where a participant answers questions several times per day for an extended period of days), to model how dynamic systems of symptoms interrelate with each other to maintain pathology, within *one person*. To date, there has been very little investigation of this method applied to treatment. The existing research finds that both patients and therapists are willing to use such individual models, though it was preferred by patients over therapists [20]. This research also found wide variation in idiographic models for individuals with anxiety and mood disorders similar to what has been found in the ED field [33–36],Levinson et al., 2021), again pinpointing the high heterogeneity present in EDs and related psychiatric illnesses, and the need for personalized treatments that address this heterogeneity.

While idiographic models have not been readily used for treatment as of yet, there is a burgeoning literature implementing this modeling technique to better conceptualize and understand how psychiatric illness maintains itself in a wide range of disorders, including EDs, anxiety, depression, and post-traumatic stress disorder [5, 8, 18, 20, 27, 29, 33–35, 41, 44]. Overall, these studies used idiographic NA to identify heterogenous individual symptom profiles, which highlights the importance of using individual networks to detect individual symptom dynamics and identify targets for precision intervention planning [5, 18, 20, 27, 41, 44]. Such a precision treatment would have high clinical utility, as a large body of research shows that clinician judgment is often biased [1, 9, 22], and in one small study idiographic data-driven treatments outperform even a team-based approach to clinical decision making [16]. In other words, a personalized treatment approach based on idiographic data could alleviate clinician pressure to have to ‘guess’ which symptom to target, instead providing a data-driven method to guide treatment planning [42].

The next step in this research is to understand how to best use idiographic NA to guide the selection of treatment targets for the development of personalized treatments. As such, the current study (*N* = 34 participants with an ED diagnosis) began to investigate how to select personalized treatment targets utilizing idiographic NA. We present data on several potential targets identified from temporal and contemporaneous networks. We use initial data collected via EMA over the course of two weeks, five times a day (75 total measurement points) that were used to select treatment targets for a personalized treatment of ED. We use this data to (a) identify a range of targets for personalized ED treatment, (b) discuss target selection issues, (c) provide guidance on how to use idiographic NA to inform treatment, including providing examples from these data on how treatment targets could inform personalized treatment, and (d) raise issues in the field that need additional research. We hypothesized that our networks would be highly heterogenous, consistent with prior ED research [33–36], and that we would identify a wide range of potential targets. We also hypothesized that target selection methods (e.g.., centrality statistic used: see **definitions in methods**; number of symptoms in model) would influence which symptoms were selected for treatment. Our ultimate goal is to provide a proof-of-concept study that can be used as a starting point to guide the field on how to develop standardized recommendations for treatment target selection for precision treatments.

## Methods

### Participants

Participants were 34 individuals with a diagnosis of an ED. The majority of participants were female (*n* = 31; 91.2%) and White (*n* = 28; 82.4%). Other ethnicities were Hispanic (*n* = 1; 2.9%), Black (*n* = 1; 2.9%), and multi-racial (*n* = 1; 2.9%) and three not reported. Age ranged from 20–57 (*M* = 34.52, *SD* = 11.11). Participant diagnoses (determined via structured clinical interview: see below) were AN-restricting (*n* = 5; 14.7%), AN-binge-purge (*n* = 2; 5.9%), BN-purging (*n* = 4; 11.8%), BN-non-purge (*n* = 3; 8.8%), BED (*n* = 9; 26.5%), atypical AN-restricting (*n* = 4; 11.7%), atypical AN-binge-purge (*n* = 2; 5.9%), atypical BN (*n* = 1; 2.9%), atypical BN-non-purging (*n* = 3; 8.8%), and atypical BED (*n* = 1; 2.9%). Average duration of illness for those who reported was 12.33 (*SD* = 6.65).

### Procedure

This study was advertised across the United States as a study for personalized treatment of an ED. Participants completed semi-structured interviews to determine inclusion/exclusion status. Inclusion criteria consisted of a current diagnosis of ED. Exclusion criteria were active suicidal intent, mania, or psychosis. All diagnoses were double-checked by the PI (CAL). There was diagnostic agreement on 98% of cases, 2% of cases were re-reviewed and discussed with four raters to reach consensus.

### Diagnostic measures

Structured Clinical Interview for DSM-5 Eating Disorder Module ( *SCID‐5‐RV -*; [17] is a semi-structured clinical interview for making standardized DSM-5 diagnoses. This study used the ED modules to determine ED diagnosis.

Mini-International Neuropsychiatric Interview 5.0 (MINI 5.0; [47] is a semi-structured interview to assess for DSM-5 diagnoses, with excellent inter-rater and test–retest reliability, and good convergent validity [46]. We used the suicidality, mania/hypomania, and psychosis modules, which were exclusion criteria.

### Ecological momentary assessment (EMA)

Participants completed assessments five times a day for 15 days (75 time points total) through EMA before beginning personalized treatment (not reported on here). Surveys lasted approximately three minutes each and participants had one hour to respond. There were 55 selected symptoms of EDs, as well as co-occurring symptoms (anxiety, depression, worry) assessed at each time point. Please see Additional file 1: Table S1, which lists all symptoms assessed via EMA. All symptoms were assessed on a 0 to 100 scale, which has been recommended for idiographic NA to improve variability and network estimation [43].

### Treatment target selection methods

Our first step was to reduce the number of symptoms to include in our idiographic models, as it is infeasible to include all symptoms assessed (i.e., 55) because of limited power. We decided a priori to select 15 items with the highest individual means out of all items because this was a large enough number to ensure we had a comprehensive model but not too large to impact estimation procedures. We also later considered selecting items with the highest variance and will discuss this decision-making process more in the discussion. Recent work, after our a-priori decision to include 15-items suggests that for temporal networks, even with a large amount of data, it is necessary to include very few items for optimal network estimation [40]. Therefore, in addition to running models with 15 items, we decided to run models with eight items to test if this improved estimation and changed target selection. How to select which symptoms to include in each individual model is of huge importance and currently no guidelines exist on how to best reduce symptom sets. As such, we will discuss this issue more in the discussion, as well as suggest guidance on what research is needed on this topic to inform clinical target selection. Descriptive statistics were computed after EMA completion before running networks.

### Imputation

Please see details in supplement on imputation. Most models were run using all available data and without imputation.

### Idiographic model estimation (N = 1)

For each individual, we estimated contemporaneous and temporal networks for both a 15-symptom and eight-symptom networks using graphicalVAR package in R [12]. For additional details on each of these types of networks please see [36] and Epskamp et al. (2017). We estimated both temporal (a directed network displaying symptoms predicting each other across 15 days, while controlling for all other symptoms in the model at the prior measurement) and contemporaneous networks (an undirected network showing how symptoms relate to each other in the same window of measurement, controlling for prior temporal relationships) for each individual. Strength centrality was calculated for contemporaneous networks, and InStrength and OutStrength centralities were calculated for directed networks using the *centralityPlot* and *centralityTable* functions in qgraph [13]. We then present the top two central symptoms for each statistic (OutStrength, InStrength, strength) as possible treatment targets, as each of these centrality indices identifies symptoms with different theoretical importance. For example, strength centrality (i.e., the sum of all the weights of all paths) provides a measurement of which symptoms are most interconnected to other symptoms within seconds, and thus have greatest potential to spread symptom activation through the network as compared to more peripheral symptoms (see “centrality hypothesis”; [45]. OutStrength (i.e., the sum of all of the weights of all outgoing paths) identifies the symptoms exerting the most influence on other symptoms, thus providing a treatment target that can have the most potential of having downstream effects on other symptoms if intervened upon, and may be thought of as the most logical theoretical based treatment target. Similiarly, InStrength (i.e., the sum of all of the weights of all incoming paths) provides a measurement of which symptoms are receiving the most input from other symptoms in the network. In-strength may not be a logical treatment target given that it represents the symptom that is impacted by most other symptoms, however; we still present data on in-strength given there is no official guidance on which centrality index should be used.

All methods, including how to label symptoms, select symptoms, and run an idiographic network, as well as code, are included in the Additional file 1. We aimed to create a user-friendly guide that can be modified for use in future research.

### Example participants

We randomly chose two participants to use as clinical examples of how selection of treatment targets could guide clinical practice. One is included in the main text and one in the Additional file 1.

## Results

### 15-symptom network

*Contemporaneous networks.* In the contemporaneous networks (Table 1), we identified 21 different most central targets and 19 different second most central targets out of 55 total symptoms. There was extremely wide variability in targets, with no one target identified as most central for most participants. The most frequently identified targets were body dissatisfaction (seven participants), drive for thinness (seven participants), and shame (five participants).

**Table 1:** Frequency of symptoms with highest and second-highest strength centrality in contemporaneous individual networks in 15-item network

Item	Highest strength	Second-highest strength	Total
	Frequency (%)		
bodydiss	1 (2.9)	6 (17.6)	7 (10.3)
drivethin	6 (17.6)	1 (2.9)	7 (10.3)
shame	2 (5.9)	3 (8.8)	5 (7.4)
worry	1 (2.9)	3 (8.8)	4 (5.9)
depression	2 (5.9)	1 (2.9)	3 (4.4)
fearlosgcntrol	2 (5.9)	1 (2.9)	3 (4.4)
fearreject	0 (0)	3 (8.8)	3 (4.4)
feelineffectve	3 (8.8)	0 (0)	3 (4.4)
saa	3 (8.8)	0 (0)	3 (4.4)
avoidemo	2 (5.9)	1 (2.9)	3 (4.4)
eatrules	1 (2.9)	1 (2.9)	2 (2.9)
excexercse	1 (2.9)	1 (2.9)	2 (2.9)
fowg	0 (0)	2 (5.9)	2 (2.9)
gad	0 (0)	2 (5.9)	2 (2.9)
physsenseat	0 (0)	2 (5.9)	2 (2.9)
selfcrit	0 (0)	2 (5.9)	2 (2.9)
binge	1 (2.9)	0 (0)	1 (1.5)
bodycheck	0 (0)	1 (2.9)	1 (1.5)
cogrestraint	1 (2.9)	0 (0)	1 (1.5)
diffeatpublic	1 (2.9)	0 (0)	1 (1.5)
diffrelax	1 (2.9)	0 (0)	1 (1.5)
eatanx	0 (0)	1 (2.9)	1 (1.5)
fearattn	0 (0)	1 (2.9)	1 (1.5)
fearmstkes	1 (2.9)	0 (0)	1 (1.5)
guilt	1 (2.9)	0 (0)	1 (1.5)
highstndrds	0 (0)	1 (2.9)	1 (1.5)
hungeranx	1 (2.9)	0 (0)	1 (1.5)
impulse	0 (0)	1 (2.9)	1 (1.5)
overvalwtshape	1 (2.9)	0 (0)	1 (1.5)
overwhelmemo	1 (2.9)	0 (0)	1 (1.5)
ptsd	1 (2.9)	0 (0)	1 (1.5)
Total	34 (100)	34 (100)	68 (100)

Top central symptoms by total percentage are bolded. See Additional file 1:Table S1 for symptom descriptions. Bodydiss = body dissatisfaction; drivethin = drive for thinness; fearlosgcntrol = fear of losing control; feelineffectve = feeling ineffective; saa = social appearance anxiety; avoidemo = avoiding emotions; eatrules = eating rules; excexercse = excessive exercise; fowg = fear of weight gain; gad = generalized anxiety disorder; physsenseat = physical sensations of eating; selfcrit = self-criticism; binge = binge eating; bodycheck = body checking; cogrestraint = cognitive restraint; diffeatpublic = difficulty eating in public; diffrelax = difficulty relaxing; eatanx = eating anxiety; fearattn = fear of attracting attention; fearmstkes = fear of making mistakes; highstndrds = high standards; overvalwtshape = overvaluation of weight and shape; overwhelmemo = overwhelming emotions; ptsd = post-traumatic stress disorder.

*Temporal Networks.* In temporal networks (Table 2) using the OutStrength statistic, we identified 17 different most central targets and 15 different second most central targets. There was wide variability in targets, though body dissatisfaction was most frequent, which was central for six participants. Using InStrength, we identified 14 different most central symptoms and 13 s most central symptoms. Fear of weight gain (five participants), anxiety about hunger (four participants), and overvaluation of weight and shape (four participants) were the most commonly identified symptoms via InStrength. Fourteen participants had no central symptoms in their temporal network, due to unidentification of autoregressive vectors from one time point to the next, meaning we needed more data and/or less items.

**Table 2:** Frequency of symptoms with the highest and second-highest centrality in temporal individual networks in 15-symptom network

Item	Highest out-strength	Second-highest out-strength	Highest in-strength	Second-highest in-strength	Total
	Frequency (%)				
bodydiss	4 (11.8)	2 (5.9)	3 (8.8)	0 (0)	9 (6.6)
fowg	1 (2.9)	1 (2.9)	3 (8.8)	2 (5.9)	7 (5.1)
hungeranx	1 (2.9)	1 (2.9)	2 (5.9)	2 (5.9)	6 (4.4)
overvalwtshape	0 (0)	2 (5.9)	1 (2.9)	3 (8.8)	6 (4.4)
diffrelax	1 (2.9)	1 (2.9)	1 (2.9)	1 (2.9)	4 (2.9)
allornothing	1 (2.9)	1 (2.9)	1 (2.9)	0 (0)	3 (2.2)
drivethin	1 (2.9)	0 (0)	1 (2.9)	1 (2.9)	3 (2.2)
eatrules	0 (0)	0 (0)	2 (5.9)	1 (2.9)	3 (2.2)
highstndrds	1 (2.9)	1 (2.9)	0 (0)	1 (2.9)	3 (2.2)
skipmeal	0 (0)	2 (5.9)	1 (2.9)	0 (0)	3 (2.2)
worry	0 (0)	1 (2.9)	0 (0)	2 (5.9)	3 (2.2)
diffeatpublic	0 (0)	0 (0)	0 (0)	2 (5.9)	2 (1.5)
fearlosgcntrol	1 (2.9)	0 (0)	1 (2.9)	0 (0)	2 (1.5)
feelineffectve	1 (2.9)	0 (0)	1 (2.9)	0 (0)	2 (1.5)
gad	0 (0)	2 (5.9)	0 (0)	0 (0)	2 (1.5)
repthghtfood	1 (2.9)	1 (2.9)	0 (0)	0 (0)	2 (1.5)
selfcrit	0 (0)	1 (2.9)	1 (2.9)	0 (0)	2 (1.5)
shame	1 (2.9)	1 (2.9)	0 (0)	0 (0)	2 (1.5)
adhd	1 (2.9)	0 (0)	0 (0)	0 (0)	1 (0.7)
compuls	0 (0)	0 (0)	0 (0)	1 (2.9)	1 (0.7)
depression	0 (0)	1 (2.9)	0 (0)	0 (0)	1 (0.7)
excexercse	0 (0)	0 (0)	0 (0)	1 (2.9)	1 (0.7)
fearatten	0 (0)	0 (0)	0 (0)	1 (2.9)	1 (0.7)
fearmstkes	0 (0)	0 (0)	0 (0)	1 (2.9)	1 (0.7)
fearreject	1 (2.9)	0 (0)	0 (0)	0 (0)	1 (0.7)
foodavoid	1 (2.9)	0 (0)	0 (0)	0 (0)	1 (0.7)
impulse	1 (2.9)	0 (0)	0 (0)	0 (0)	1 (0.7)
iuc	1 (2.9)	0 (0)	0 (0)	0 (0)	1 (0.7)
mealrum	0 (0)	1 (2.9)	0 (0)	0 (0)	1 (0.7)
postevprocess	1 (2.9)	0 (0)	0 (0)	0 (0)	1 (0.7)
ruminate	0 (0)	0 (0)	1 (2.9)	0 (0)	1 (0.7)
sleepdiff	0 (0)	0 (0)	1 (2.9)	0 (0)	1 (0.7)
NA	14 (41.2)	15 (44.1)	14 (41.2)	15 (44.1)	58 (42.6)
Total	34 (100)	34 (100)	34 (100)	34 (100)	136 (100)

NA = no central symptom identified. Top central symptoms by total percentage are bolded. See Additional file 1: Table S1 for symptom descriptions. Bodydiss = body dissatisfaction; fowg = fear of weight gain; hungeranx = hunger anxiety; overvalwtshape = overvaluation weight and shape; diffrelax = difficulty relaxing; allornothing = all-or-nothing perfectionism; drivethin = drive for thinness; eatrules = eating rules; highstndrds = high standards; skipmeal = skipping meals; diffeatpublic = difficulty eating in public; fearlosgcntrol = fear of losing control; feelineffectve = feeling ineffective; gad = generalized anxiety disorder; repthghtfood = repetitive thoughts about food; selfcrit = self-criticism; adhd = attention deficit/hyperactivity disorder; compuls = compulsions; excexercse = excessive exercise; fearatten = fear attracting attention; fearmstkes = fear of making mistakes; fearreject = fear of rejection; food avoid = food avoidance; impulse = impulsivity; iuc = intolerance of uncertainty; mealrum = meal rumination; postevprocess = post-event processing; ruminate = rumination; sleepdiff = sleep difficulties

### Eight-symptom network

#### Contemporaneous networks

In the contemporaneous networks (Table 3), we identified 22 different most central targets and 19 different second most central targets. There was extremely wide variability in targets, with no one target identified as most central for most participants. The most frequently identified targets were body dissatisfaction (seven participants), drive for thinness (seven participants), and fear of weight gain (five participants).

**Table 3:** Frequency of Symptoms with Highest and Second-Highest Centrality in Contemporaneous Individual Networks in 8-Symptom Network

Item	Highest strength	Second-highest strength	Total
	Frequency (%)		
bodydiss	4 (10.8)	3 (9.4)	7 (10.1)
drivethin	3 (8.1)	4 (12.5)	7 (10.1)
fowg	2 (5.4)	3 (9.4)	5 (7.2)
saa	1 (2.7)	3 (9.4)	4 (5.8)
selfcrit*	3 (8.1)	1 (3.1)	4 (5.8)
cogrestraint*	3 (8.1)	0 (0)	3 (4.3)
gad	2 (5.4)	1 (3.1)	3 (4.3)
shame**	2 (5.4)	1 (3.1)	3 (4.3)
worry	1 (2.7)	2 (6.3)	3 (4.3)
bodycheck	2 (5.4)	0 (0)	2 (2.9)
depression	0 (0)	2 (6.3)	2 (2.9)
eatrules	1 (2.7)	1 (3.1)	2 (2.9)
fearmstkes	0 (0)	2 (6.3)	2 (2.9)
feelineffectve**	1 (2.7)	1 (3.1)	2 (2.9)
guilt	0 (0)	2 (6.3)	2 (2.9)
impulse	1 (2.7)	1 (3.1)	2 (2.9)
overvalwtshape	2 (5.4)	0 (0)	2 (2.9)
skipmeal*	2 (5.4)	0 (0)	2 (2.9)
adhd	0 (0)	1 (3.1)	1 (1.4)
compuls	0 (0)	1 (3.1)	1 (1.4)
diffeatpublic	1 (2.7)	0 (0)	1 (1.4)
diffrelax	1 (2.7)	0 (0)	1 (1.4)
excexercse	1 (2.7)	0 (0)	1 (1.4)
fearlosgcntrol	0 (0)	1 (3.1)	1 (1.4)
fearreject	1 (2.7)	0 (0)	1 (1.4)
highstndrds	1 (2.7)	0 (0)	1 (1.4)
interoaware	1 (2.7)	0 (0)	1 (1.4)
iuc*	1 (2.7)	0 (0)	1 (1.4)
mealrum	0 (0)	1 (3.1)	1 (1.4)
repthghtfood	0 (0)	1 (3.1)	1 (1.4)
Total	37 (100)	32 (100)	69 (100)

*For three participants, the highest and second-highest strength had the same value, so both symptoms were listed under the highest strength. For two of these participants, the symptoms with the same value were skipmeal and cogrestraint. For one of these participants, the symptoms with the same value were iuc and selfcrit. **For one participant, the second-highest and third-highest strength had the same value, so both symptoms (feelineffectve and shame) were listed under second-highest strength. Top central symptoms by total percentage are bolded. See Additional file 1: Table S1 for symptom descriptions. Body diss = body dissatisfaction; drivethin = drive for thinness; fowg = fear of weight gain; saa = social appearance anxiety; selfcrit = self-criticism, cogrestraint = cognitive restraint; gad = generalized anxiety disorder; bodycheck = body checking; eatrules = eating rules; fearmstkes = fear of making mistakes; feelineffectve = feeling ineffective; impulse = impulsivity; overvalwtshape = overvaluation of weight and shape; skipmeal = skipping meals; adhd = attention-deficit/hyperactivity disorder; compuls = compulsions; diffeatpublic = difficulty eating in public; diffrelax = difficulty relaxing; excexercse = excessive exercise; fearlosgcntrol = fear of losing control; fearreject = fear of rejection; highstndrds = high standards; interoaware = interoceptive awareness; iuc = intolerance of uncertainty; mealrum = meal rumination; repthghtfood = repetitive thoughts about food.

#### Temporal networks

In temporal networks (Table 4) using the OutStrength statistic we identified 14 different most central targets and 16 different second most central targets. Again, there was wide variability in targets, though body dissatisfaction and drive for thinness were most frequent, each as most central for four participants. Using InStrength we identified 19 different most central targets and 15 s most central targets. Body dissatisfaction (six participants), fear of weight gain (five participants), and drive for thinness (four participants) were the most commonly identified targets via in-strength. Ten participants had no central symptoms in their temporal network.

**Table 4:** Frequency of symptoms with the highest and second-highest centrality in temporal individual networks in 8-symptom network

Item	Highest out-strength	Second-highest out-strength	Highest in-strength	Second-Highest In-Strength	Total
	Frequency (%)				
bodydiss	4 (11.8)	0 (0)	3 (8.8)	3 (8.8)	10 (7.4)
drivethin	3 (8.8)	1 (2.9)	1 (2.9)	3 (8.8)	8 (5.9)
fowg	0 (0)	1 (2.9)	4 (11.8)	1 (2.9)	6 (4.4)
overvalwtshape	2 (5.9)	0 (0)	1 (2.9)	2 (5.9)	5 (3.7)
cogrestraint	2 (5.9)	1 (2.9)	1 (2.9)	0 (0)	4 (2.9)
eatrules	0 (0)	2 (5.9)	1 (2.9)	1 (2.9)	4 (2.9)
foodavoid	1 (2.9)	2 (5.9)	1 (2.9)	0 (0)	4 (2.9)
selfcrit	2 (5.9)	1 (2.9)	1 (2.9)	0 (0)	4 (2.9)
feelineffectve	0 (0)	2 (5.9)	1 (2.9)	0 (0)	3 (2.2)
hungeranx	1 (2.9)	0 (0)	1 (2.9)	1 (2.9)	3 (2.2)
binge	0 (0)	1 (2.9)	1 (2.9)	0 (0)	2 (1.5)
bodycheck	1 (2.9)	1 (2.9)	0 (0)	0 (0)	2 (1.5)
diffeatpublic	1 (2.9)	1 (2.9)	0 (0)	0 (0)	2 (1.5)
diffrelax	1 (2.9)	0 (0)	0 (0)	1 (2.9)	2 (1.5)
fearlosgcntrol	0 (0)	1 (2.9)	0 (0)	1 (2.9)	2 (1.5)
highstndrds	0 (0)	0 (0)	1 (2.9)	1 (2.9)	2 (1.5)
impulse	1 (2.9)	0 (0)	1 (2.9)	0 (0)	2 (1.5)
saa	0 (0)	2 (5.9)	0 (0)	0 (0)	2 (1.5)
shame	0 (0)	1 (2.9)	1 (2.9)	0 (0)	2 (1.5)
skipmeal	2 (5.9)	0 (0)	0 (0)	0 (0)	2 (1.5)
sleepdiff	0 (0)	1 (2.9)	0 (0)	1 (2.9)	2 (1.5)
socialintanx	2 (5.9)	0 (0)	0 (0)	0 (0)	2 (1.5)
worry	0 (0)	0 (0)	1 (2.9)	1 (2.9)	2 (1.5)
adhd	0 (0)	0 (0)	1 (2.9)	0 (0)	1 (0.7)
avoidemo	0 (0)	0 (0)	1 (2.9)	0 (0)	1 (0.7)
eatanx	0 (0)	0 (0)	0 (0)	1 (2.9)	1 (0.7)
excexercse	0 (0)	0 (0)	1 (2.9)	0 (0)	1 (0.7)
fearmstkes	0 (0)	0 (0)	1 (2.9)	0 (0)	1 (0.7)
fearreject	0 (0)	0 (0)	0 (0)	1 (2.9)	1 (0.7)
gad	0 (0)	1 (2.9)	0 (0)	0 (0)	1 (0.7)
guilt	1 (2.9)	0 (0)	0 (0)	0 (0)	1 (0.7)
mealrum	0 (0)	1 (2.9)	0 (0)	0 (0)	1 (0.7)
physsenseat	0 (0)	0 (0)	0 (0)	1 (2.9)	1 (0.7)
ruminate	0 (0)	0 (0)	0 (0)	1 (2.9)	1 (0.7)
NA	10 (29.4)	14 (41.2)	10 (29.4)	14 (41.2)	48 (35.3)
Total	34 (100)	34 (100)	34 (100)	34 (100)	136 (100)

NA = no central symptoms identified. Top central symptoms by total percentage are bolded. See Additional file 1: Table S1 for symptom descriptions. Bodydiss = bodydissatisfaction; drivethin = drive for thinness; fowg = fear of weight gain; overvalwtshape = overvaluation weight of shape; cogrestraint = cognitive restraint; eatrules = eating rules; foodavoid = food avoidance; selfcrit = self-criticism; feelineffectve = feeling ineffective; hungeranx = hunger anxiety; binge = binge eating; bodycheck = body checking; diffeatpublic = difficulty eating in public; diffrelax = difficulty relaxing; fearlosgcntrol = fear of losing control; highstndrds = high standards; impulse = impulsivity; saa = social appearance anxiety; skipmeal = skipping meals; sleediff = sleep difficulties; socialintanx = social interaction anxiety; adhd = attention deficit/hyperactivity disorder; avoidemo = avoiding emotions; eatanx = eating anxiety; excexercse = excessive exercise; fearmstkes = fear of making mistakes; fearreject = fear of rejection; gad = generalized anxiety disorder; mealrum = meal rumination; physsenseat = physical sensations of eating; ruminate = rumination

### Networks

**Fig. 1.**
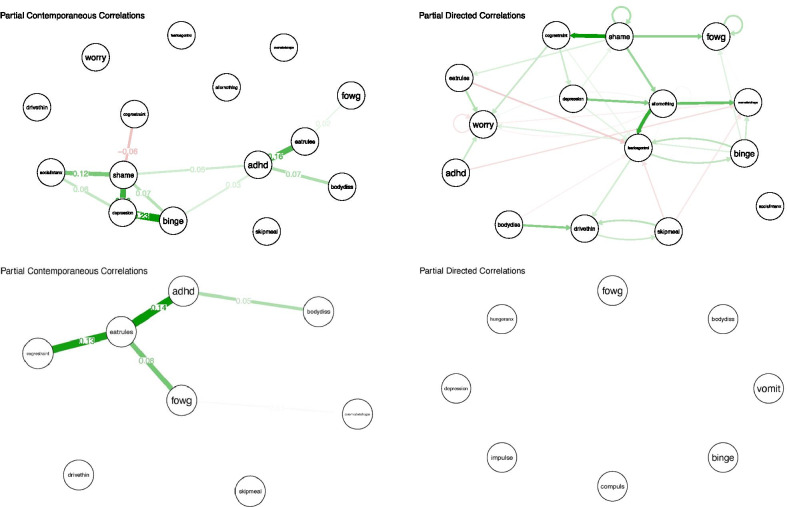
15-item (top) and 8-item (bottom) contemporaneous (left) and temporal (right) individual networks for example participant. See Additional file 1: Table S1 for full items associated with each node abbreviation

**Fig. 2.**
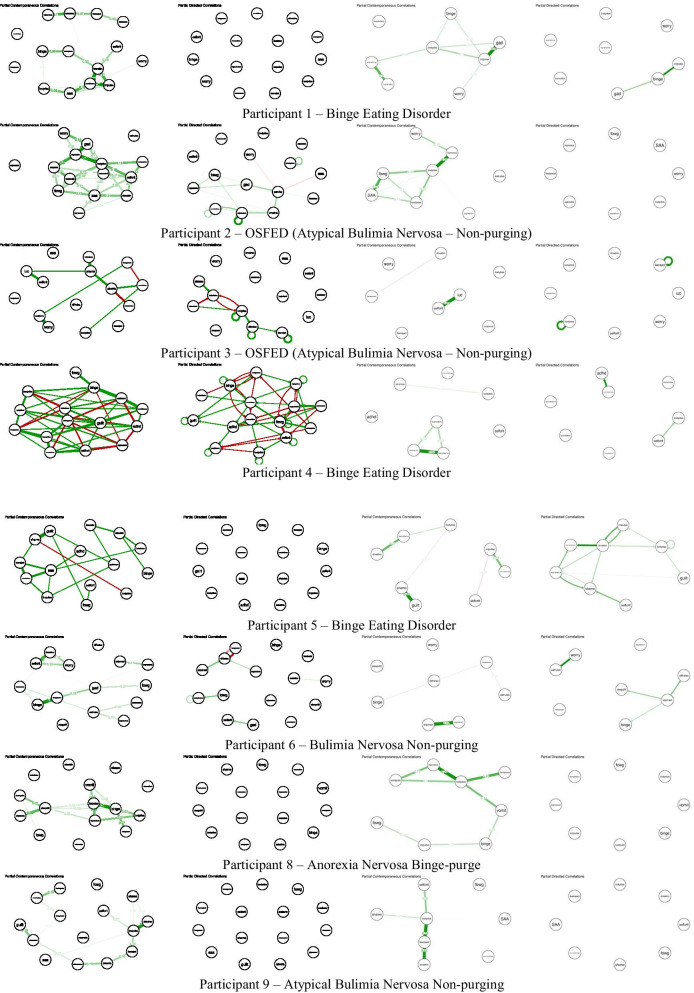

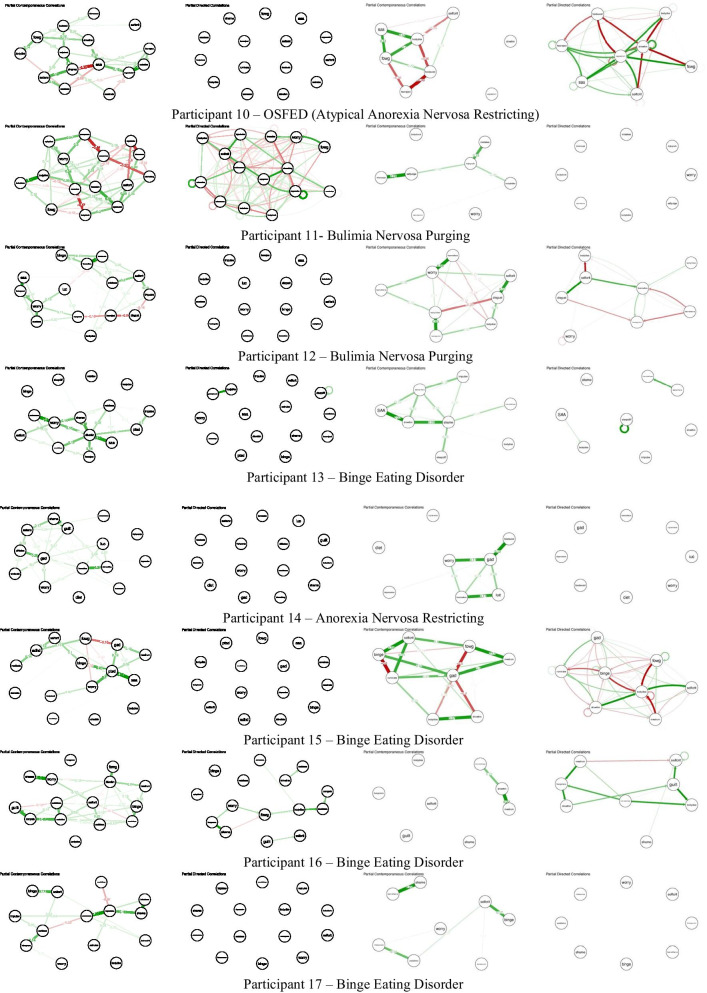

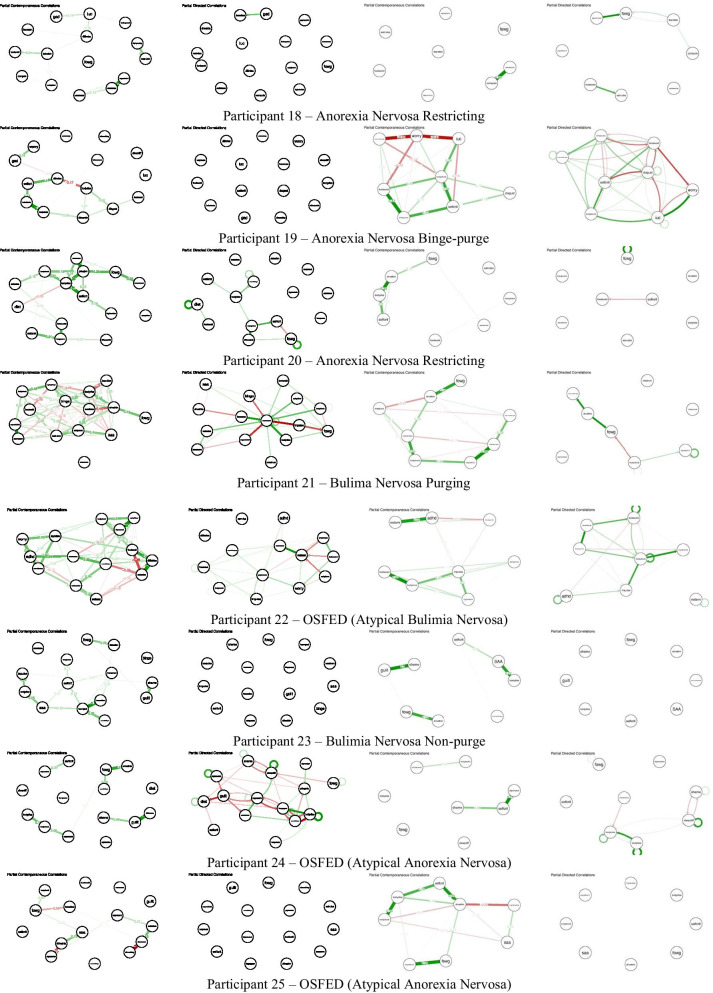

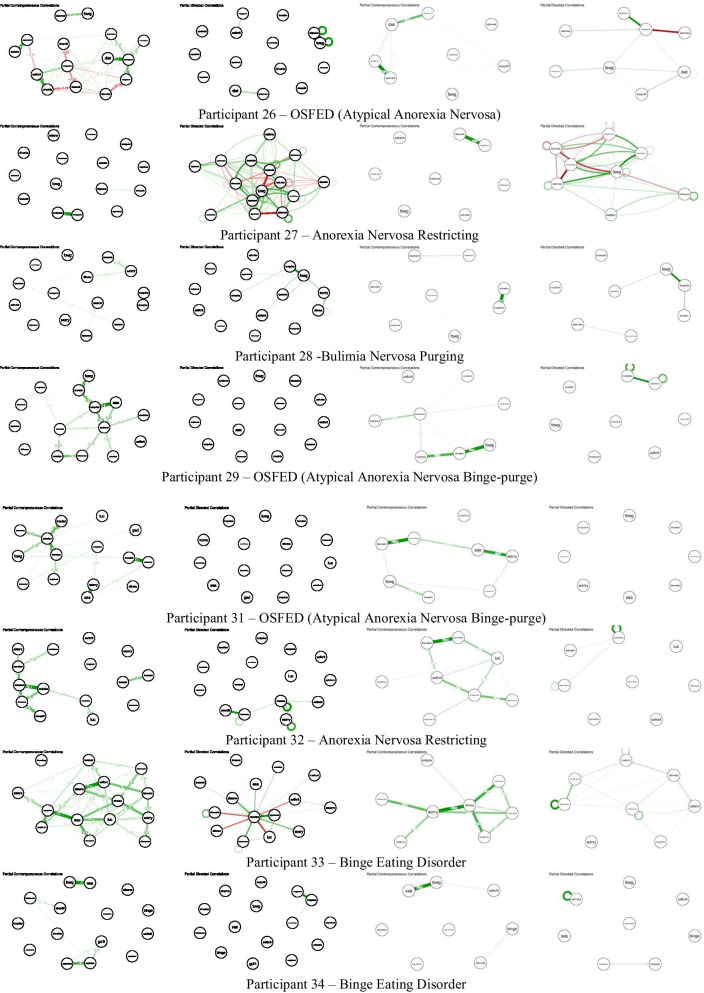
15-item and 8-item contemporaneous and temporal individual networks for additional participants. See Figs. 1/S1 for networks of participants 7 and 30. See Additional file 1: Table S1 for full items associated with each node abbreviation

Please see Fig. 1/S1 for networks of example participants. Please see Fig. 2 for all other participant networks.

### Example participants

We use two example participants, randomly selected, from the eight-symptom networks to illustrate how intervention might occur based on the selected targets and relations shown in the network. These examples are meant to illustrate how clinicians could use the temporal and contemporaneous centrality statistics to guide which treatment modules they apply to patients and how these treatments would significantly vary from treatment as usual. Participant 7 is described here, and Participant 30 can be found in the Additional file 1.

### Participant 7

*Temporal Targets.* The two strongest temporal out-strength symptoms are drive for thinness and feeling ineffective; the two strongest temporal in-strength symptoms are shame and overvaluation of weight and shape. Based on the out-strength and in-strength symptoms, a clinician would focus on intervening primarily on the two symptoms identified via out-strength, as they are theorized to be key in the maintenance of other symptoms by providing the most input to other symptoms out of all other symptoms in the model. For drive for thinness and feeling ineffective, treatment could utilize specific modules from CBT-E focused on drive for thinness and feeling ineffective before completing other CBT-E modules. Specifically, a clinician could identify thinking errors, automatic thoughts, and core beliefs; challenge automatic thoughts; and use behavioral experiments to challenge the individuals’ core beliefs [14]. If the clinician wanted to focus on in-strength symptoms, for shame, a clinician could use emotion regulation from dialectical behavior therapy (DBT; [39] and focus on exploring feelings of shame, how to check the facts, and engaging in opposite action. Finally, for overvaluation of weight and shape, treatment again could use modules from CBT-E and would focus on identifying thinking errors and automatic thoughts, challenging automatic thoughts, values assessment and exploration, and goal setting. Based on the partial directed correlations network for this individual, there are several relationships these interventions should disrupt. First, drive for thinness leads to increased overvaluation of weight and shape, feeling ineffective, and shame at the next time point. Further, there is a relationship between drive for thinness and impulsivity, such that each lead to an increase in the other at the next time point. Feeling ineffective leads to increased overvaluation of shape and weight and guilt at the next time point and there is a relationship between feeling ineffective and shame, such that each lead to an increase in the other at the next time point.

*Contemporaneous Targets.* Alternately, for this individual the two strongest contemporaneous symptom targets are shame and guilt, therefore, if this statistic was used treatment would be very different. Although shame was captured as one of the strongest in-strength temporal targets, guilt is a new intervention target for this individual based on the contemporaneous symptom relationships. For both shame and guilt, a clinician could use emotion regulation from DBT as described in the *Temporal Targets* section above for this individual. Of course, all of these suggestions for treatments are suggestions only and clinicians could use their own expertise and training to decide which treatments to apply to these targets once identified.

## Discussion

The current research is a proof-of-concept study illustrating how idiographic NA can be used to select treatment targets for personalized treatment. We used data from 34 participants with an ED to show how symptom assessment, symptom selection, model type and statistic can influence which targets are selected. We provided clinical examples of how individual treatment targets could influence treatment planning and ordering of interventions even if drawn from the same treatment. Overall, we found that, as hypothesized, ED symptom profiles were highly heterogeneous. No one target was identified as most important, rather there was a wide range of targets selected, highlighting why a personalized treatment method is needed. Such a method could help overcome barriers to delivering evidence-based effective ED care by addressing both high heterogeneity and reduced reliance on clinician-judgment alone.

### Eating disorder personalized treatment targets

We found a wide variety of potential ED treatment targets. Out of the 55 symptoms assessed, depending on the model and statistic, we found between 13–22 different treatment targets. No single target was overwhelmingly found to be most important. There were not discernible differences by diagnosis, which has also been found prior [33–36] meaning that regardless of diagnosis there is high heterogeneity. The most frequently occurring most central symptoms (i.e. targets) were body dissatisfaction, drive for thinness, and fear of weight gain, regardless of model type or statistic. This finding is consistent with growing literature showing that overvaluation of weight and shape, fear of weight gain, and related constructs are most central ‘on average’ for individuals with EDs [11, 19, 33–35, 37, 49]. However, we want to emphasize that the combination of weight and shape related constructs (drive for thinness, body dissatisfaction, fear of weight gain) were central in less than half of participants. Many other participants had most central targets such as shame, feeling ineffective, fearing losing control, and anxiety. This finding is similar to treatment response rates of around 50% [30]. In just under 50% of individuals, overvaluation of weight and shape was central, which is the main target mechanism of treatments such as CBT-E (in addition to a reduction in ED behaviors: [15]. It may be possible these similar rates are coincidental- though future research is needed to test if personalization for those without a central shape and weight concern improves treatment. For individuals who have a target not related to overvaluation of weight and shape, it makes theoretical sense why such a treatment may not be as effective, as additional, yet important targets may be left untreated (or never targeted) until later in treatment reducing the efficacy of treatment. For these individuals, it seems likely that addressing their specific central symptoms either as primary treatment targets or earlier in treatment could lead to more effective or shorter treatment. This hypothesis remains yet to be tested with empirical data.

We also want to emphasize that while overvaluation of weight and shape related symptoms were central in just under half of the identified targets, there were several different aspects of overvaluation that were endorsed. Specifically, we found participants had individual targets focused on body dissatisfaction, drive for thinness, fear or weight gain, and overvaluation of weight and shape: each of which are different constructs/symptoms [4, 38]. The implication of this finding is that even when targeting overvaluation of weight and shape, the treatment may need to be tailored to focus on different aspects of weight and shape concerns in different individuals. For example, the treatment of fear of weight gain, via imaginal exposure, may be warranted for some individuals, whereas cognitive restructuring or acceptance-based treatments may be warranted for symptoms such as body dissatisfaction and drive for thinness [2, 24, 33–35]. Currently, treatments such as imaginal exposure for fear of weight gain, are not included in CBT-E.

### Treatment target selection issues

There are several issues that we encountered that are worth mentioning. We hope that researchers will reflect on these issues, as well as how to use them to create standard guidance on how to select treatment targets using idiographic modeling. In this discussion, we have attempted to pinpoint areas that are in need of future research. Ultimately, our goal is to showcase these issues so that they can be used as a starting point to guide the field on how to develop standardized recommendations for treatment target selection for precision treatments. We also want to emphasize that while we do not yet have all the answers for how to best personalize treatment, this dilemma does not reflect an opinion that personalized treatment via NA cannot be effective; rather our goal is to understand how to make it *most* effective.

#### Assessment

First, which symptoms should be included in assessment? We sought to assess as many symptoms as feasible via EMA to ensure that we had a comprehensive picture of ED symptoms and co-occurring conditions. This research can be used as a starting point, such that symptoms we found to be central in any individual should conclusively be included in future assessment. However, it is entirely possible there are symptoms that we did not assess that are important mechanisms/intervention targets for EDs. There is also the issue of topological overlap. For example, the distinction between shape and weight concerns. The field as a whole could benefit from a comprehensive collection of possible ED symptoms to include in momentary assessment used for personalized treatment. Further, there are several possible methods that could improve assessment. Given that it is not feasible to collect all possible symptoms, nor to model all symptoms, an idiographic assessment approach may be needed. For example, participants might answer all symptom questions at the beginning of the assessment period and then if there are symptoms that are not endorsed, these symptoms would no longer be assessed [23]. Additionally, there is currently work that is integrating clinician and patient report with data [6] to develop network models for idiographic target selection. It seems plausible to us that clinicians and/or patients themselves may have important insight into which symptoms should be assessed and included in a comprehensive patient-clinician-data model. Further, there are no standardized recommendations for how many assessment points are needed, with the primary recommendation to collect as much data as is feasible. Last, psychoeducation to participants on the importance of utilizing the entire scale range is important, as well as education on what the rating anchors mean, to ensure that participants answer accurately and do not select, for example, all 100s, which then limits variance and impacts model estimation.

#### Item inclusion

Once symptoms have been assessed, there are logistical problems of selecting which items to include in the model. As with any model, there must be a balance between comprehensiveness and parsimony. Recent work suggests that for temporal networks, even with a large amount of data, it is necessary to include very few items for optimal network estimation [40]. However, the inability to include a large number of symptoms limits the comprehensiveness of our models and increases the likelihood we will miss important symptoms. In our data, we showed models that included 15 symptoms and eight symptoms. While we were able to identify more temporal targets for more people in the eight-item models, there was still a large proportion that did not have central symptoms in the temporal models, possibly because of limits of the analytic technique and the need for more data. Thus, the number of symptoms in the model balanced with the amount of data available is clearly an important consideration.

Relatedly, the decision of what number of symptoms to include a-priori is of utmost significance. In our data we used the symptoms with the top eight or 15 highest means across 15 days (75 data points). However, it has also been suggested that using the symptoms with the highest variance might also be a way to select symptoms (personal communication, S. Epskamp, 2020). The highest means versus most variant symptoms will have different implications for the model and both intensity and variation in symptoms has been shown to influence pathology [25, 26]. Research is needed to test which of these item selection methods is most clinically relevant. First, does intervention on mean vs. variant selected symptoms produce better treatment outcomes? Second, do one of these specific target selection methods identify targets that better predict clinical outcomes? Or is there another method to select symptoms that could outperform both of these strategies? It may be that a combination of highest mean and variant symptoms may ultimately be the best solution. Clearly, much future research is needed.

*Type of centrality statistic.* We presented three different types of centrality statistics from two idiographic models: temporal (out and in-strength) and contemporaneous strength centrality. While there was some convergence across statistic type in the central symptom selected, there was also variation, as would be expected from models based on different theoretical assumptions. While temporal out-strength (the amount of output a symptom gives to all other symptoms in the model) may seem to be the logical candidate for ideal target, these are also the models that need the most data. Relatedly, based on theory, in-strength is the least likely centrality statistic that should be used to select treatment targets because these are symptoms that are *receiving* rather than *giving* input [45]. It also seems plausible to us that contemporaneous central symptoms could be treatment targets, given that how symptoms operate in the span of seconds may also be important for the maintenance of pathology. No research exists comparing selected targets from each of these different types of models as predictors of clinical outcomes or treatment response. In the current study (see Example participants), we illustrate how treatment would proceed based on treatment targets from each of these different models/statistics. For example, if there are different targets identified via out-strength (temporal) vs strength (contemporaneous), the types of treatments that correspond to these symptoms would be different and encompass an entirely dissimilar treatment plan. Future research is needed to compare treatments selected via different centrality metrics. Finally, we did not include other centrality indices, such as controllability and expected influence, which should also be considered as potential statistics that could be translated to treatment targets, especially given negative edges in networks (Henry, Robinaugh, & Fried, 2020).

*Number of treatment targets.* Last, in the current study we selected the top two central symptoms as our potential treatment targets. Guidance on how many central symptoms should be selected for intervention does not exist. It is possible that intervention on one symptom could produce clinically significant change. However, it is also entirely possible that the number of central symptoms could vary based on illness and person. For example, some patients may need to have treatment focused on several targets, whereas others may only need one treatment target. For example, it is possible that if fear of weight gain is the identified treatment target, participants would only need treatment (imaginal exposure) on that target. Whereas for an individual who has central symptoms of binge eating and purging, they may need intervention on both of those symptoms. Future research is needed to explore this issue and develop guidance on how many symptoms should be selected as targets.

### Clinical implications

Despite the plethora of unanswered questions regarding idiographic modeling methodologies for the development of personalized treatments, we think this research holds great promise for clinical practice. We believe that the combination of idiographic methodologies with user-friendly software can make this type of approach highly feasible in clinical settings, pending future research on dissemination and implementation. More research is needed on how to use idiographic modeling in a format that is acceptable for clinicians, to help bridge the research-practice gap.

From the current study, our results show that less than half of individuals with an ED, regardless of model or statistic, endorse overvaluation of weight and shape or related symptoms as most central and that there were several aspects of overvaluation of weight and shape that were central for different individuals. This finding is consistent with response rates for CBT-E and encourages us that we are on the right track toward development of treatments that will be inclusive for all symptom presentations. While this data does not yet make clear exactly how to *best* personalize treatment, it does show clearly *why* treatments developed based on averages do not work for a substantial percentage of patients and highlights the dire need for personalized treatments based in data. If a treatment is designed to address a symptom that is not important for maintaining pathology, it follows that the treatment would not work and must be modified to address the symptom of importance, and will likely improve the speed and efficiency of treatment response. It is possible that these types of methods might be used with all patients to personalize treatment or that non-responders to typical evidence-based treatments might particularly benefit.

### Future research

In addition to the practical issues raised above, there are two main areas in need of future research and several additional future research directions. First, these models are data, time, and analytic intensive and must be made translatable to clinicians without those areas of expertise. If a disseminated personalized treatment for ED (and other psychiatric illnesses) is the goal, idiographic scientists must work with clinicians, computer programmers, and engineers to develop a user-friendly system that can interpret idiographic models and is easy for clinicians to use and integrate into treatment and takes into account clinician input. Second, most of our treatments to date are designed based on categorical disorders. For example, there are treatments for EDs, generalized anxiety disorder, unipolar depression, etc. NA inherently uses a different approach, in which symptoms take the forefront, instead of categories. Unfortunately, this means that most problematic symptoms (e.g., emotional avoidance, hunger anxiety) do not have a specific intervention that is evidence-based. Researchers have been able to work around this issue by using evidence-based protocols such as the Unified Protocol [16]. However, specifically for the EDs, there is very little research on treatment modules that can be matched to specific target *symptoms*. This type of treatment development work is necessary if a network-informed personalized treatment target selection approach proves effective. In addition, future research should consider testing how idiographic networks change pre to post treatment, if they are impacted by duration/stage of illness, both of which may help inform how symptoms operate as mechanisms during treatment, as well as inform dynamic, just-in-time-interventions. Future research should continue to establish the best ways in which to select measurement via EMA and strive for a standardized set of measures both within the ED field and across all areas of psychiatric research. It would also be interested to test non-linear dynamic relationships. Finally, future research should continue to consider theory in the expansion of network models (see Fried, 2020 for a discussion), given the natural overlap between network theory and theoretical orientations such as CBT and DBT.

## Conclusions

We presented initial data from a proof-of-concept study with 34 participants with an ED enrolled in a personalized treatment trial. We found that there was a wide variety in central symptoms that could be used as treatment targets. Just under half of our participants endorsed weight and shape related symptoms, whereas the large majority had a wide range of central symptoms including cognitions, behaviors, affect, and co-occurring disorders. These rates are consistent with response rates for evidence-based treatments for EDs, which show about 50% of patients respond and suggests that personalized treatments that target individual maintenance factors are needed. There are many remaining issues that idiographic scientists need to understand to create evidence-based personalized treatments and this proof-of-concept study highlights areas in need of more research, as well as suggests a need for standardized recommendations across the idiographic network field. Crucially, the establishment of evidence-based personalized treatments for EDs, as well as for a wide range of related psychiatric illnesses, holds great promise for the improvement of response rates and reduction of suffering that accompanies this deadly disorder.

## Supplementary Information

**Additional file 1.** Supplemental Material.

## Data Availability

The datasets analyzed during the current pilot study are not publicly available due to being pilot data of an ongoing trial but will be made available from the corresponding author upon completion of the full study.

## Abbreviations

AN: Anorexia nervosa
BED: Binge eating disorder
BN: Bulimia nervosa
CBT-E: Enhanced cognitive behavioral therapy
DBT: Dialectical behavior therapy
ED: Eating disorder
EMA: Ecological momentary assessment
NA: Network analysis
OSFED: Other specified feeding and eating disorders
PTSD: Post-traumatic stress disorder

## References

[1] Ægisdóttir S, White MJ, Spengler PM, Maugherman AS, Anderson LA, Cook RS, Nichols CN, Lampropoulos GK, Walker BS, Cohen G, Rush JD (2006). The meta-analysis of clinical judgment project: fifty-six years of accumulated research on clinical versus statistical prediction. Couns Psychol.

[2] Agras WS (2019). Cognitive behavior therapy for the eating disorders. Psychiatr Clin North Am.

[3] Arcelus J (2011). Mortality rates in patients with anorexia nervosa and other eating disorders: a meta-analysis of 36 studies. Arch Gen Psychiatry.

[4] Askew AJ, Peterson CB, Crow SJ, Mitchell JE, Halmi KA, Agras WS, Haynos AF (2020). Not all body image constructs are created equal: Predicting eating disorder outcomes from preoccupation, dissatisfaction, and overvaluation. Int J Eat Disord.

[5] Bringmann LF, Ferrer E, Hamaker EL, Borsboom D, Tuerlinckx F (2018). Modeling nonstationary emotion dynamics in dyads using a time-varying vector-autoregressive model. Multivar Behav Res.

[6] Burger J, A Bayesian Approach to Incorporating Clinical Expertise in Personalized Symptom Networks 2020.

[7] Carter JC, Mercer-Lynn KB, Norwood SJ, Bewell-Weiss CV, Crosby RD, Woodside DB, Olmsted MP (2012). A prospective study of predictors of relapse in anorexia nervosa: Implications for relapse prevention. Psychiatry Res.

[8] David SJ, Marshall AJ, Evanovich EK, Mumma GH (2018). Intraindividual dynamic network analysis – implications for clinical assessment. J Psychopathol Behav Assess.

[9] Dawes RM, Faust D, Meehl PE. Clinical Versus Actuarial Judgment. 1989;8. 10.1126/science.2648573 2648573

[10] Deloitte Access Economics. Social and economic cost of eating disorders in the United States of America. Academy for Eating Disorders 2020.

[11] DuBois RH, Rodgers RF, Franko DL, Eddy KT, Thomas JJ (2017). A network analysis investigation of the cognitive-behavioral theory of eating disorders. Behav Res Ther.

[12] Epskamp S, Borsboom D, Fried EI (2018). Estimating psychological networks and their accuracy: A tutorial paper. Behav Res Methods.

[13] Epskamp S, Cramer AOJ, Waldorp LJ, Schmittmann VD, Borsboom D, qgraph: Network visualizations of relationships in psychometric data. J Stat Softw, 2012;48(1), 1–18. 10.18637/jss.v048.i04

[14] Fairburn CG (2008). Cognitive behavior therapy and eating disorders.

[15] Fairburn CG, Cooper Z, Shafran R (2003). Cognitive behaviour therapy for eating disorders: A “transdiagnostic” theory and treatment. Behav Res Ther.

[16] Fernandez KC, Fisher AJ, Chi C (2017). Development and initial implementation of the dynamic assessment treatment algorithm (DATA). PLoS ONE.

[17] First MB, Williams JBW, Karg RS, Spitzer RL, Structured clinical interview for DSM-5 – research version (SCID-5 for DSM-5, research version; SCID-5-RV). American Psychiatric Association, 2015

[18] Fisher AJ, Reeves JW, Lawyer G, Medaglia JD, Rubel JA (2017). Exploring the idiographic dynamics of mood and anxiety via network analysis. J Abnorm Psychol.

[19] Forbush KT, Siew CSQ, Vitevitch MS (2016). Application of network analysis to identify interactive systems of eating disorder psychopathology. Psychol Med.

[20] Frumkin MR, Piccirillo ML, Beck ED, Grossman JT, Rodebaugh TL (2020). Feasibility and utility of idiographic models in the clinic: a pilot study. Psychother Res.

[21] Galmiche M, Déchelotte P, Lambert G, Tavolacci MP (2019). Prevalence of eating disorders over the 2000–2018 period: a systematic literature review. Am J Clin Nutr.

[22] Garb HN (2005). Clinical judgment and decision making. Annu Rev Clin Psychol.

[23] Gibbons RD, Weiss DJ, Frank E, Kupfer D (2016). Computerized adaptive diagnosis and testing of mental health disorders. Annu Rev Clin Psychol.

[24] Griffiths C, Williamson H, Zucchelli F, Paraskeva N, Moss T (2018). A systematic review of the effectiveness of acceptance and commitment therapy (ACT) for body image dissatisfaction and weight self-stigma in adults. J Contemp Psychother.

[25] Hamaker EL, In Why researchers should think “within-person”: a paradigmatic rationale: Mehl MR, Conner TS., Handbook of research methods for studying daily life. (p. 43.61). The Guilford Press 2012.

[26] Houben M, Van Den Noortgate W, Kuppens P (2015). The relation between short-term emotion dynamics and psychological well-being: A meta-analysis. Psychol Bull.

[27] Howe E, Bosley HG, Fisher AJ (2020). Idiographic network analysis of discrete mood states prior to treatment. Couns Psychother Res.

[28] Insel TR (2014). The NIMH research domain criteria (RDoC) project: precision medicine for psychiatry. Am J Psychiatry.

[29] Kaiser T, Laireiter A-R, Process-symptom-bridges in psychotherapy: An idiographic network approach. J Person Orient Res, 2018;49–62. 10.17505/jpor.2018.06 10.17505/jpor.2018.06 PMC7842641 33569133

[30] Keel PK, Brown TA (2010). Update on course and outcome in eating disorders. Int J Eat Disord.

[31] Keel PK, Mitchell JE (1997). Outcome in bulimia nervosa. Am J Psychiatry.

[32] Keel PK, Dorer DJ, Franko DL, Jackson SC, Herzog DB (2005). Postremission predictors of relapse in women with eating disorders. Am J Psychiatry.

[33] Levinson CA, Cash E, Welch K, Epskamp S, Hunt RA, Williams BM, Keshishian AC, Spoor SP (2020). Personalized networks of eating disorder symptoms predicting eating disorder outcomes and remission. Int J Eat Disord.

[34] Levinson CA, Christian C, Ram SS, Vanzhula I, Brosof LC, Michelson LP, Williams BM (2020). Eating disorder symptoms and core eating disorder fears decrease during online imaginal exposure therapy for eating disorders. J Affect Disord.

[35] Levinson CA, Vanzhula IA, Smith TW, Stice E (2020). Group and longitudinal intra-individual networks of eating disorder symptoms in adolescents and young adults at-risk for an eating disorder. Behav Res Ther.

[36] Levinson CA, Vanzhula I, Brosof LC (2018). Longitudinal and personalized networks of eating disorder cognitions and behaviors: targets for precision intervention a proof of concept study. Int J Eat Disord.

[37] Levinson CA, Zerwas S, Calebs B, Forbush K, Kordy H, Watson H, Hofmeier S, Levine M, Crosby RD, Peat C, Runfola CD, Zimmer B, Moesner M, Marcus MD, Bulik CM (2017). The core symptoms of bulimia nervosa, anxiety, and depression: a network analysis. J Abnorm Psychol.

[38] Levitt DH (2003). Drive for thinness and fear of fat: separate yet related constructs?. Eat Disord.

[39] Linehan MM, Cognitive-behavioral treatment of borderline personality disorder (1st ed.). Guilford Publications 1993.

[40] Mansueto AC, Wiers R, Weert JCM van, Schouten BC, Epskamp S, Investigating the feasibility of idiographic network models. 2020. 10.31234/osf.io/hgcz6 10.1037/met0000466 34990189

[41] Moeller J, Ivcevic Z, Brackett MA, White AE (2018). Mixed emotions: Network analyses of intra-individual co-occurrences within and across situations. Emotion.

[42] Piccirillo ML, Beck ED, Rodebaugh TL (2019). A clinician’s primer for idiographic research: considerations and recommendations. Behav Ther.

[43] Piccirillo ML, Rodebaugh TL (2019). Foundations of idiographic methods in psychology and applications for psychotherapy. Clin Psychol Rev.

[44] Reeves JW, Fisher AJ (2020). An examination of idiographic networks of posttraumatic stress disorder symptoms. J Trauma Stress.

[45] Robinaugh DJ, Hoekstra RHA, Toner ER, Borsboom D (2020). The network approach to psychopathology: a review of the literature 2008–2018 and an agenda for future research. Psychol Med.

[46] Sheehan DV, Lecrubier Y, Sheehan KH, Janavs J, Weiller E, Keskiner A, Schinka J, Knapp E, Sheehan MF, Dunbar GC (1997). The validity of the Mini International Neuropsychiatric Interview (MINI) according to the SCID-P and its reliability. Eur Psychiatry.

[47] Sheehan DV, Lecrubier Y, Sheehan KH, Amorim P, Janavs J, Weiller E, Hergueta T, Baker R, Dunbar GC (1998). The mini-international neuropsychiatric interview (MINI): the development and validation of a structured diagnostic psychiatric interview for DSM-IV and ICD-10. J Clin Psychiatry.

[48] Strober M, Freeman R, Morrell W (1997). The long-term course of severe anorexia nervosa in adolescents: survival analysis of recovery, relapse, and outcome predictors over 10–15 years in a prospective study. Int J Eat Disord.

[49] Wang SB, Jones PJ, Dreier M, Elliott H, Grilo CM (2019). Core psychopathology of treatment-seeking patients with binge-eating disorder: a network analysis investigation. Psychol Med.

[50] Wilson GT, Grilo CM, Vitousek KM (2007). Psychological treatment of eating disorders. Am Psychol.

[51] Wright AG, Woods WC (2020). Personalized models of psychopathology. Annual Rev Clin Psychol.

