# Correction to: Using individual networks to identify treatment targets for eating disorder treatment: a proof-of-concept study and initial data

**DOI:** 10.1186/s40337-022-00626-6

**Published:** 2022-07-11

**Authors:** Cheri A. Levinson, Rowan A. Hunt, Ani C. Keshishian, Mackenzie L. Brown, Irina Vanzhula, Caroline Christian, Leigh C. Brosof, Brenna M. Williams

**Affiliations:** Department of Psychological and Brain Sciences, Life Sciences Building, University of Louisville, Louisville, KY 40292 USA

## Correction to: J Eat Disord (2021) 9:147 https://doi.org/10.1186/s40337-021-00504-7

Unfortunately, the original version of this article [1] contained an error with reference to the example participants’ numbers.

In the original version, the example participants’ numbers were given as participant 7 and participant 30. However, the in-text references refer to network figures of participants 5 and 21.

The correct figures for participant 5 and 21 are shown below.
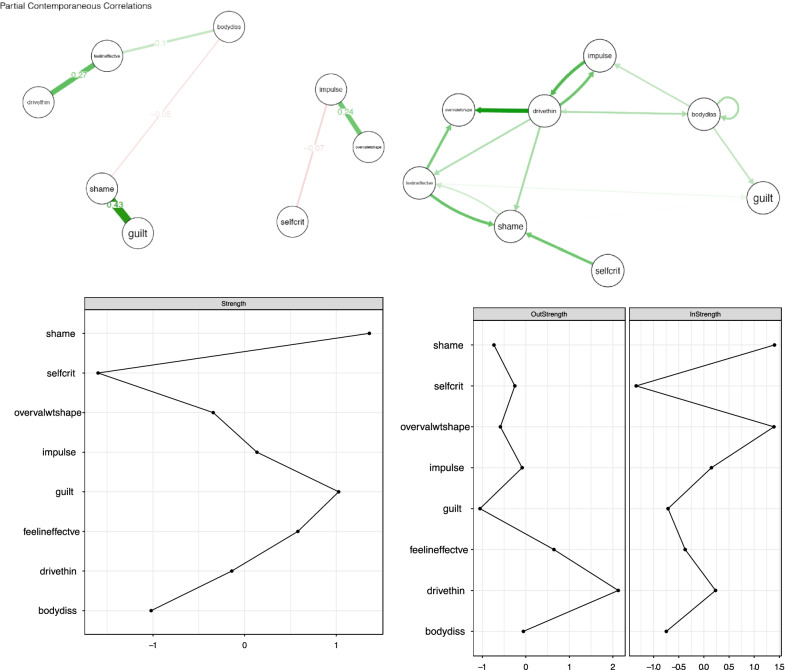



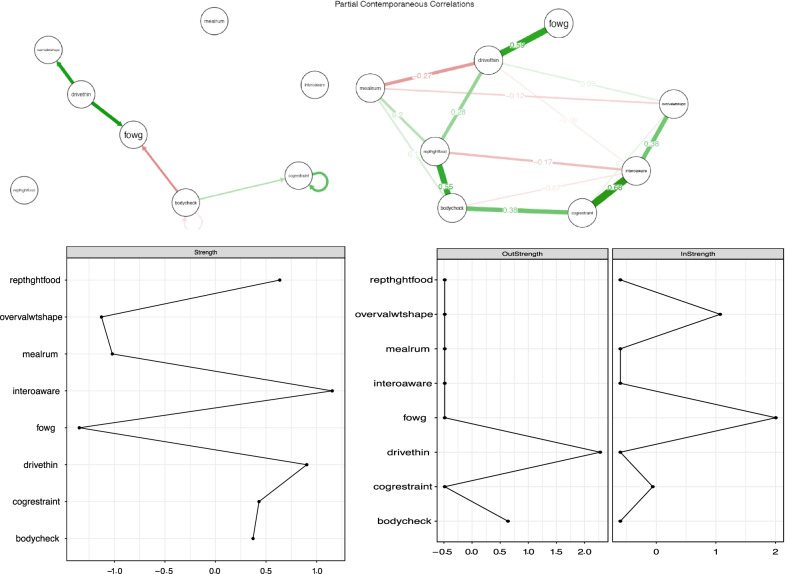


## References

[1] Levinson (2021). Using individual networks to identify treatment targets for eating disorder treatment: a proof-of-concept study and initial data. J Eat Disord.

