# Supplementary material for: Using individual networks to identify treatment targets for eating disorder treatment: a proof-of-concept study and initial data
Source: J Eat Disord. 2021 Nov 4;9:147. doi: 10.1186/s40337-021-00504-7 (PMC8567590; doi:10.1186/s40337-021-00504-7)
Supplement: Supplementary file 1 — Additional file 1. Supplemental Material. [file 40337_2021_504_MOESM1_ESM.docx]

**Supplemental Material**

**Idiographic Model Estimation for Use in Personalized Treatment**

1. Review your patient’s experience sampling data regularly. If they are using the same numbers a lot, email them a reminder to use the entire scale (0 to 100). If they have multiple days with same high numbers on multiple variables (several 100s or 90s), you may need to ask your patient to complete a few more days of EMA to ensure more variability for the data analysis.
2. When EMA is complete
   1. Download file from RedCap
   2. Clean the data, code missing data
   3. Run descriptive statistics for all variables (mean, SD, range)
   4. Copy and paste descriptives table in Excel format into Excel file
   5. Sort all variables by mean from highest to smallest
   6. Identify 15 items with highest means
   7. Check variability of items (range and SD)
3. Create excel file with only top 15 variables
   1. Replace -99 with NA
   2. Add day, beep, and time variables
4. Run the network using code below. Identify top 2 symptoms with highest centrality
   1. **NOTE: please use standardized network names for symptoms (see end of document**
5. Use contemporaneous network to choose 2 top symptoms
6. If you get error messages, you may not have enough variability. In this case you will:
   1. Collect more data (and attempt running network again)
   2. If collecting more data is not feasible, you will then **impute the 15 item dataset** and attempt running the network with the imputed dataset.
   3. If the network still does not run, discuss with team how to proceed.
7. Select an evidence-based treatment for these top 2 symptoms

**EMA Items**

Part of the goal of this study was to identify the entire range of possible treatment targets for EDs so we assessed 55 possible symptoms. These symptoms were selected based on a large literature review completed for the current study to identify any possible maintaining symptom and/or transdiagnostic feature (e.g., perfectionism, low self-worth) of EDs and/or co-occurring condition and were discussed with eight experts/trainees in the ED field. We then drew symptom assessments from existing momentary surveys, self-report questionnaires, or in some instances, developed new items when there was no existing measure. Symptom categories for assessment were selected based on the cognitive-behavioral transdiagnostic theory of EDs (Fairburn et al., 2003) and the high comorbidity of EDs with other psychiatric illnesses (Pallister & Waller, 2008). Thus, symptoms fall into four categories, behaviors (*n* = 16), cognitions (n = 19), affect (*n* = 13), and co-occurring conditions (n = 7).

**Imputation**

Participants were missing between 0-83% of data, with an average of 27.2% missing data per each participant. Models were first estimated with missing data retained. If a model would not converge, more data was collected from participants when feasible for up to 75 data points. For those whom further data collection was not feasible (e.g., participant was unable to complete more assessment due to technology issues), or who still had non-converging models after additional data collection, their data was imputed (*n* = 6 participants) using multiple imputation by chained equations using the mice package in R (version 3.4.3; Buuren & Groothuis-Oudshoorn, 2011) and the predictive mean modeling method to provide necessary stability.

**Participant 30**

*Temporal Targets.* For this individual, the two strongest temporal out-strength symptoms are drive for thinness and body checking; the two strongest temporal in-strength symptoms are fear of weight gain and overvaluation of weight and shape. Again, treatment would most likely focus on the two symptoms with the highest out-strength given this is the type of centrality that assesses how symptoms impact the most other symptoms. To target drive for thinness, again we could rely on modules from CBT-E. This treatment would need to specifically be modified to also focus on body checking. Treatment modules would include self-monitoring body checking and prevention and habit-breaking of both body checking and other appearance rituals. If a clinician wanted to focus on the in-strength targets, for fear of weight gain, treatment would focus on imaginal exposure for fear of weight gain (Levinson et al., 2020). Finally, for overvaluation of weight and shape, treatment could again rely on modules from CBT-E.

*Contemporaneous Targets.* Alternately, for this individual the two strongest contemporaneous symptom targets are interoceptive awareness and drive for thinness. Although drive for thinness was captured as one of the strongest out-strength temporal targets, interoceptive awareness is a new intervention target for this individual based on the contemporaneous symptom relationships. For drive for thinness, as described in the *Temporal Targets* section above, modules could be used from CBT-E. However, to target interoceptive awareness, a clinician could utilize exposure therapy. Specifically, a clinician could work with the individual to create a hierarchy of interoceptive awareness and conduct exposures designed to illicit uncomfortable physical sensations and to teach the patient how to sit with these sensations.

Table S1. Symptoms Assessed via Experience Sampling and Used to Model Idiographic Networks

| Symptom Category | Symptom | Symptom Abbreviation |
| --- | --- | --- |
| Cognitions |  |  |
|  | Fear of weight gain | fowg |
|  | Drive for thinness | drivethin |
|  | Overvaluation of weight and shape | overvalwtshape |
|  | Fear of rejection | fearreject |
|  | Fear of making mistakes | fearmstkes |
|  | All-or-nothing perfection | allornothing |
|  | High standards | highstndrds |
|  | Intolerance of uncertainty | iuc |
|  | Social appearance anxiety | SAA |
|  | Worry | worry |
|  | Rumination | ruminate |
|  | Post-event processing | postevprocess |
|  | Self-criticism | selfcrit |
|  | Obsessions | obsess |
|  | Fear of losing control | fearlosgcntrol |
|  | Body dissatisfaction | bodydiss |
|  | Feeling ineffective | feelineffectve |
|  | Repetitive thoughts about food | repthghtfood |
|  | Meal Rumination | mealrum |
| Behaviors |  |  |
|  | Skipping meals | skipmeal |
|  | Cognitive restraint | cogrestraint |
|  | Dieting | diet |
|  | Eating rules | eatrules |
|  | Self-induced vomiting | vomit |
|  | Laxative use | laxative |
|  | Diuretic use | diuretic |
|  | Binge eating | binge |
|  | Excessive exercise | excexercse |
|  | Body checking | bodycheck |
|  | Compulsions | compuls |
|  | Sleep difficulties | sleepdiff |
|  | Difficulty drinking in public | diffdrinkpublic |
|  | Difficulty eating in public | diffeatpublic |
|  | Impulsivity | impulse |
|  | Food avoidance | foodavoid |
| Affect/Emotion |  |  |
|  | Guilt | guilt |
|  | Shame | shame |
|  | Depression | depression |
|  | Avoiding emotions | avoidemo |
|  | Overwhelming emotions | overwhlmemo |
|  | Difficulty identifying emotions | diffidentemo |
|  | Eating anxiety | eatanx |
|  | Disgust | disgust |
|  | Difficulty relaxing | diffrelax |
|  | Heart racing | hrtrace |
|  | Interoceptive awareness | interoaware |
|  | Physical sensations of eating | physsenseat |
|  | Hunger anxiety | hungeranx |
| Co-occurring conditions |  |  |
|  | Post-traumatic stress disorder | ptsd |
|  | Obsessive compulsive disorder | ocd |
|  | Social interaction anxiety | socialintanx |
|  | Fear of attracting attention | fearatten |
|  | Generalized anxiety disorder | gad |
|  | Attention deficit/hyperactivity disorder | adhd |
|  | Alcohol and substance use | alcsubuse |

*Note.* All symptoms were assessed on a 0 to 100 scale, for 15 days, 5 times a day.

|  |  |
| --- | --- |
| 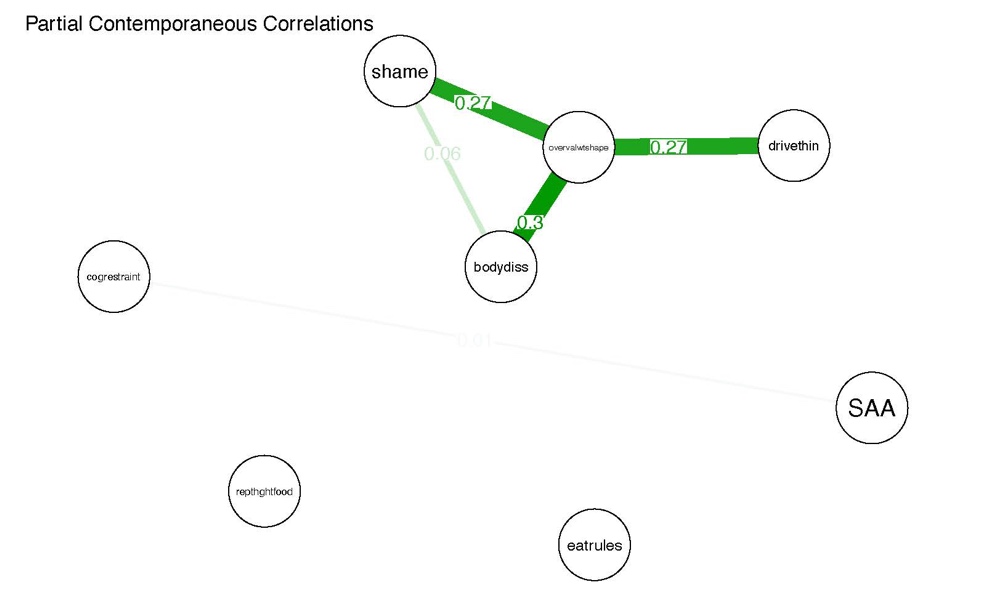 | 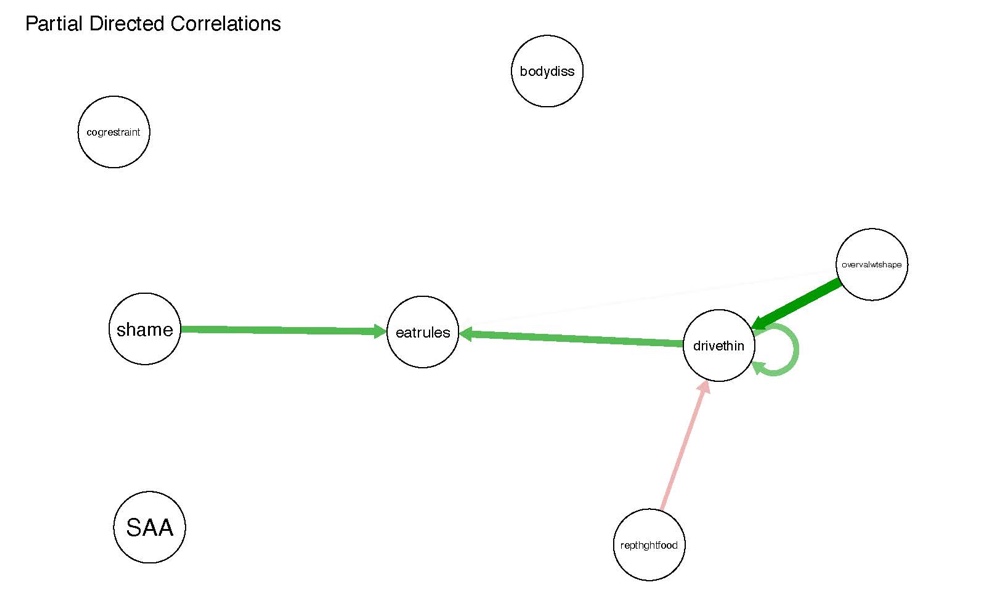 |
| Participant 30 - Bulimia Nervosa Non-purging | |

*Figure S1.* 15-item (top) and 8-item (bottom) contemporaneous (left) and temporal (right) individual networks for example participant. See supplemental Table S1 for full items associated with each node abbreviation.

**Network Code**

library(readxl)

library(graphicalVAR)

library(qgraph)

data <- as.data.frame(read_excel("C:/Users/lapab/Dropbox/Personalized Treatment Study/Data and Networks/PT001/pt001reduced4.xlsx",na = "NA"))

Vars <- c("thinner", "thinkself", "reject", "mistakes", "highgoal", "judge", "worry", "disapprove", "dislike", "avoid", "guilty", "ashamed", "depressed", "emotions", "overwhelm") ###*make sure these variables match your top 15*

beepvar <- "beep"

dayvar <- "day"

data$time <- as.POSIXct(data$time) #read as time data

data$Day <- as.Date(data$time)

N1 <-graphicalVAR(data, vars=Vars, dayvar=dayvar, beepvar=beepvar, gamma=0)

plot(N1, nodeNames = Vars, layout = "spring")

pdf("pt001.pdf", height=5, width=8)

u1 <- plot(N1, "PCC", labels = Vars, edge.labels = TRUE, layout="spring")

u2 <- plot(N1, "PDC", labels = Vars)

dev.off()

pdf("pt001-1.pdf", height=5, width=8)

u1 <- plot(N1, "PCC", labels = Vars, edge.labels = TRUE, layout="spring")

dev.off()

pdf ("u1-2")

c1 <- centralityPlot(u1)

c2 <- centralityPlot(u2)

dev.off()

c2 <- centralityTable(u1)

write.csv(c2, "personcentrality.csv")
